# Expression, purification and functionality of bioactive recombinant human vascular endothelial growth factor VEGF_165_ in *E. coli*

**DOI:** 10.1186/s13568-016-0300-2

**Published:** 2017-02-06

**Authors:** Awatef Taktak-BenAmar, Maram Morjen, Hazem Ben Mabrouk, Rania Abdelmaksoud-Dammak, Mohamed Guerfali, Najla Fourati-Masmoudi, Naziha Marrakchi, Ali Gargouri

**Affiliations:** 1 Laboratoire de Biotechnologie Moléculaire des Eucaryotes, Centre de Biotechnologie de Sfax, University of Sfax, BP1177, 3018 Sfax, Tunisia; 2 Laboratoire des Venins et Biomolécules Thérapeutiques, LR11IPT08, Institut Pasteur de Tunis, Université de Tunis el Manar, 13, Place Pasteur, 1002 Tunis, Tunisia; 3 Service Analyse, Centre de Biotechnologie de Sfax, University of Sfax, BP1177, 3018 Sfax, Tunisia

**Keywords:** RT-PCR, Soluble VEGF_165_ expression, Inclusion bodies, Refolding, Purification, Cell migration and proliferation, CAM assay

## Abstract

Vascular endothelial growth factor (VEGF) is associated with tumour growth and metastasis. Because VEGF is the major player in both angiogenesis and vascular permeability and the most explored factor in angio-inhibitory therapies, many expression procedures have been developed to produce functional VEGF_165_ in convenient yield. In this study, recombinant human VEGF_165_ was cloned and expressed in *Escherichia coli* (BL21)-DE3 cells and large scale production was performed by fermentation. A high yield of active soluble protein was obtained after protein extraction employing both lysozyme and sonication treatment. Inclusion bodies were also isolated from the cell lysate and subjected to a simple protocol of solubilisation and refolding. Single-step purification was performed using nickel affinity chromatography and the purified proteins were able to recognize monoclonal Anti-poly-His antibody. The biological activity of the VEGF_165_ was successfully tested using the Chicken chorioallantoic membrane assay, wound-healing migration and proliferation assay on human umbilical vein endothelial cells (HUVEC).

## Introduction

Angiogenesis is considered as a complex multistep process involving the growth of blood vessels from the existing vasculature (Adair and Montani 2010). Formation of new blood vessels can takes place under both normal physiological conditions such as embryonic development, endometrial and placental proliferation, growth and tissue repair, as well as pathological ones, including cancer vascularization. The promotion of tumour growth is dependent on the expression of growth factors in the microenvironment like vascular endothelial growth factor (VEGF), heparin-binding fibroblast growth factor (FGF), and platelet-derived endothelial cell growth factor (PD-ECGF) (Niu and Chen 2010). VEGF ranks as key inducer of angiogenesis and central mediator that promotes vascular permeability (Schmitz et al. 2006). Several proteins including VEGF-A to D and placental growth factor (PlGF) compose the VEGF family. They do not share high homology but they share cysteine “knot motif” comprising eight conserved cysteine residues. VEGF-A binds to VEGFR-1 and -2, mediating the activation of all pathways required in angiogenesis. VEGF-A which commonly referred to as VEGF, was firstly isolated in 1989 from medium conditioned by bovine pituitary follicular cells (Ferrara and Henzel 1989) and described in highly vascularized tumours where its expression is stimulated by hypoxia (Shweiki et al. 1992). The VEGF pre-mRNA is transcribed from a single gene containing 8 exons, is spliced and expressed as various isoforms owing to alternative splicing of exon 6 and 7. These two exons determine the VEGF fate, either being associated to cell surface or being secreted and associated to the extracellular matrix (Roodink and Leenders 2010). There are at least 4 principal variants VEGF_121_, VEGF_165_, VEGF_189_ and VEGF_206_ with the numerals denoting the number of amino acids in the mature peptide (Roskoski 2007; Ferrara et al. 1992).

VEGF_165_, the most abundant isoform with VEGF_121_, is secreted and represents the most relevant promoter of tumour vascularization as it exerts several effects in different pathways required in angiogenesis such as endothelial cell migration, proliferation, tube formation and survival (Papetti and Herman 2002) and is therefore the focus of intense investigation. It has been reported that the quantification of the total VEGF mRNA expression by real-time reverse transcription PCR revealed that VEGF_121_ and VEGF_165_ mRNA were up-regulated in various neoplasm compared to normal tissue (Zygalaki et al. 2007; Hervé et al. 2008). VEGF_121_ and VEGF_165_ were also found to be the most over-expressed isoforms in both colonic and lung carcinoma (Cheung et al. 1998). VEGF_189_ and VEGF_206_ are poorly secreted and are essentially cell associated although their peptide signal sequence is identical to that found in VEGF_121_ and VEGF_165_ (Houck et al. 1991).

In 1971, Folkman suggested the idea that anti-angiogenic therapies could be used as a highly promising and effective approach in cancer treatment (Folkman 1971). VEGF_165_ showing strong mitogenic potency to vascular endothelial cells is used to direct therapy in a wide range of cancers. On the basis of this pioneering hypothesis, numerous studies were carried out to provide a fast and easy way to produce this therapeutic protein. VEGF is a highly conserved disulfide-bonded glycoprotein with a molecular mass of 43 kDa consisting of an anti-parallel homodimer structure (Vicari et al. 2011). The VEGF belonging to the PDGF family is characterized by the presence of eight conserved cysteine residues implicated in intra- and inter-chain disulfide bonds (Keyt et al. 1996).

Since the functional potency of VEGF_165_ is not dependent on the N-linked glycosylation at Asn75 residue, eukaryotic expression platform is not required for VEGF recombinant protein production (Claffey et al. 1995). Many bacterial expression systems have been developed to achieve high yield as well as high quality and functional potency of the VEGF_165_. It was generally reported that expression resulted in most of cases in the formation of inclusion bodies which represented the primary source of the expressed protein (Gast et al. 2011). *Escherichia coli* which remains one of the most attractive cell hosts, have been widely utilized for production of recombinant His-tagged proteins.

In the current study, we report on soluble His-tagged VEGF_165_ protein that was successfully expressed in *E. coli* (BL21)-DE3. Key factors for efficient production were assessed and optimization of cell growth conditions and media were sought. Several practical methods have been implemented to ensure high cell-density cultivation. Our methods allow us to consistently obtain high yield of biological active VEGF_165_. More importantly, different protein extraction procedures with optimized conditions were performed to achieve high solubility of the expressed protein. An economically and fast protein extraction protocol combining sonication and lysozyme treatment was used to facilitate soluble VEGF_165_ extraction. Moreover, expression of VEGF_165_ in *E. coli* (BL21)-DE3 often results in accumulation of the recombinant protein as insoluble aggregates. We describe here an economic and efficient process for solubilisation and refolding of the VEGF_165_ aggregates.

## Materials and methods

### Strains and culture conditions

pGEMT-easy and pET-21a (+) vectors were used respectively to clone and to express the VEGF splice variants proteins. *E. coli* strains Top10 (*F*-*mcrAΔ(mrr*-*hsdRMS*-*mcrBC) φ80lacZΔM15 ΔlacX74 nupG recA1 araD139 Δ(ara*–*leu)7697 galE15 galK16 rpsL(StrR) endA1 λ*-*)* and BL21(DE3) *(F*–*ompT gal dcmlonhsdSB(rB*-*mB*-*) λ(DE3 [lacI lacUV5*-*T7 gene 1 ind1 sam7 nin5])* were used as recipient for cloning and expression vectors respectively.

Culture media were LB (Luria-Bertani):10 g/l bacto-tryptone, 5 g/l yeast extract, 5 g/l NaCl; 2YT: 17 g/l bacto-tryptone, 10 g/l yeast extract, 5 g/l NaCl. LBA and 2YTA: LB and 2YT containing 100 µg/ml ampicillin.

The VEGF_165_ coding sequence, isolated from MCF7 cell lines, was 100% identical to the human VEGF_165_, already published (Piotrowski et al. 2015) under the accession number NM_001287044.1, was cloned downstream of the T7*lac* promoter and transformed into *E. coli* BL21-(DE3). Recombinant strains were cultivated on 2YTA medium and induced by the addition of 1 mM of IPTG at 37 °C for 20 h.

### Amplification of VEGF splices variants

Polymerase chain reaction (PCR) was performed with a common forward primer F1 located in exon 2 and a common reverse primer R1 located in exon 8; both exon 2 and exon 8 are parts of the conserved region of all VEGF splice variants (Table 1). R1 primer contains at its end six His residues followed by a stop codon. These primers were designed to amplify the coding region of all VEGF isoforms and to contain restriction endonuclease sites (*Bam*HI and *Xho*I) for sub-cloning into pET21a vectors. The target sequences (see “Results” section) were amplified in a 25 µl reaction volume containing either 1 µl of each cDNA (already available in our laboratory) or 1 µl of diluted Plasmid DNA (after cloning into pGEMT-easy vector), 0.2 µM of each primer, 200 µM dNTP, 1X Dream Taq PCR Buffer and 1 unit of Dream Taq Polymerase. Amplification was carried out in a DNA thermocycler (Biometra) with initial denaturation at 94 °C for 5 min, followed by 37 cycles of 30 s denaturation at 94 °C, annealing for 30 s at 60 °C, extending for 40 s at 72 °C and a final cycle of 7 min extension at 72 °C. The PCR products were analysed by electrophoresis on a 2% agarose gel that was subsequently visualized under UV illumination after ethidium bromide staining.

**Table 1:** Primers used in this work

DNA used	Primers
pGEMT easy vector	**Universal** 5′GTTTTCCCAGTCACGACGTTGTA3′ **Reverse** 5′AGCGGATAACAATTTC3′
cDNA	**F** (ex2) 5′GGATCCGCACCCATGGCAGAAGGAGGA **R** (ex8) 5′CTCGAGTCAGTGGTGGTGGTGGTGGTGCCGCCTCGGCTTGTCACATCT

### Cloning and DNA sequencing of RT-PCR products

The PCR fragment, amplified on cDNA from MCF7 cell line, was firstly cloned into the pGEMT-easy vector (Promega). Ligation product was transformed into competent *E. coli* Top10 cells and plated on LBA. A fraction (1/20) of each colony-plasmid was amplified with Dream Taq DNA polymerase using F1 and R1 primers. As these primers can detect all VEGF splice variants, each variant was identified by PCR screening and was verified by DNA sequencing using universal and reverse primers (Table 1). Thereafter, the VEGF_165_ variant was sub-cloned in pET-21a using the *Bam*HI and *Xho*I restriction sites.

### Fermentation of the recombinant strain expressing the VEGF_165_

A 7 l stirred tank bioreactor (Infos, AG GH-4103 Bottmingen, Switzerland) equipped with air flow, temperature, dissolved oxygen concentration, pH and agitation control was utilized to produce elevated levels of VEGF_165_. The fermentation was carried out with a working volume of 4 l. In the batch cultivation, the temperature was maintained at 37 °C and dissolved oxygen was kept above 20% of medium saturation by air supply and agitation rate variation (400–600 rpm). To decrease foam production Silicone 426 R antifoam (Prolabo, Paris, France) was added. The initiated pH of the medium was 7 and the pO_2_ was 98%. After 7 h, pO_2_ decreased to 43%. When the OD_600nm_ of the culture reached 0.7, IPTG was added at a final concentration of 1 mM. The induction phase was maintained for more than 16 h. After 16 h, pO_2_ continue to decrease to 1%. When pO_2_ increased to 9% (18 h of induction phase) the growth rate was found to slow down. At this pO_2_, the growth of *E. coli* cells was stopped and the cell pellet was collected by centrifugation at 6000 rpm for 20 min and stored at −20 °C.

### Cell disruption

In order to optimize the protocol of extraction of the recombinant VEGF_165_ from the cell pellet, five methods were adopted: M1: Alumina treatment; M2: lysozyme treatment; M3: sonication in PBS; M4: sonication in Lysis Buffer; M5: lysozyme treatment followed by sonication in lysis Buffer treatment. Induced cultures were centrifuged at 6000 rpm for 20 min for each of these methods.

A mechanical cell shearing performed by the abrasive effect of the alumina powder was adopted in M1. In that case, after pelleting, cells frozen at −20 °C (1.5 g) and subsequently thawed in a chilled mortar were grinded energetically for 15 min with Alumina powder w/w (Sigma-Aldrich, Munich, Germany) using a pestle until the mixture formed a fairly stiff paste (Hughes 1950). The cell paste was re-suspended in 2 ml of PBS 1X buffer containing 2 mM PMSF. Alumina, unbroken cells as well as cell debris were decanted after a low speed centrifugation at 3000 rpm for 10 min and the supernatant (crude lysate) was saved.

Osmotic lysis of bacterial cells was adopted in M2. After washing with buffer A, containing 500 mM sucrose, 25 mM Tris–HCl pH 8, 10 mM EDTA, the cell pellet was re-suspended in this hypertonic solution and then treated with lysozyme (5 mg/ml). Protoplasts were harvested by centrifugation at 4000 rpm for 10 min and burst with an osmotic imbalance created by an hypotonic solution (buffer B) composed of 25 mM Tris–HCl pH 8, then phenyl methyl-sulfonyl fluoride (PMSF) was added. The remaining non-burst cells and cells debris were centrifuged at 3000 rpm for 10 min.

Additionally, a high operating pressure using a “Vibra cell VCX 750 sonicator was performed for 30 min at 60% amplitude with PBS in M3 and with lysis buffer composed of 50 mM Tris–HCl pH 8, 150 mM NaCl, 2 mM EDTA, 1% Triton, 1% PMSF in M4.

Alternatively, combining sonication and lysozyme treatment was carried out in M5 by first treating the pelleted protoplasts with lysis buffer (50 mM Tris–HCl pH 8, 150 mM NaCl, 2 mM EDTA, 1% Triton, 1% PMSF) and then sonication for 30 min at 60% amplitude. The resulting protein extract was centrifuged at 3000 rpm for 10 min to remove unbroken cells and cell debris.

For all extraction protocols, after the mild centrifugation (3000 rpm for 10 min) the supernatant, called crude lysate, is clarified by centrifugation at 13,000 rpm for 10 min. The supernatant represented the total soluble protein extract while the pellet represented the IB (inclusion bodies).

### Inclusion bodies isolation

The IB pellet material collected from the large scale production (700 ml of culture) was predominantly used for VEGF_165_ solubilisation and refolding experiments. It was re-suspended in 50 ml of buffer containing 50 mM Tris-HCl pH 8, 50 mM NaCl, 1 mM EDTA, 1% Triton X-100. After washing with the same buffer but without Triton X-100, the inclusion bodies were collected by centrifugation at 8000 rpm for 10 min and subsequently subjected to the solubilisation step by adding of 25 ml of 8M urea and 5% β-mercaptoethanol (βME). The suspension was stirred overnight at 4 °C and then centrifuged at 8000 rpm at 4 °C for 10 min. The solubilized proteins were then dialyzed at 4 °C against 2 l of buffer containing 25 mM Tris pH8, 50 mM NaCl. The dialysis buffer was changed four times to sufficiently allow VEGF_165_ refolding. The remaining insoluble material was eliminated by centrifugation at 8000 rpm for 10 min.

### VEGF_165_ purification

A nickel affinity chromatography was used to purify the VEGF proteins. The total protein extract (either from total soluble proteins or from solubilised IB) was loaded on His Trap™chelating HP 1 ml column (GE healthcare life sciences) with a flow rate of 1 ml/min. The resin was washed with 30 ml binding buffer (20 mM NaH_2_PO_4_ Na_2_HPO_4_, 500 mM NaCl, 10 mM Imidazole, pH 7.4) to enable elution of non-specifically-bound proteins. Finally, the His-tagged proteins were eluted from the resin with Imidazole linear gradient from 10 to 500 mM in Elution Buffer (20 mM NaH_2_PO_4_ Na_2_HPO_4_, 500 mM NaCl, pH 7.4). The purity of collected fractions was assessed by SDS-PAGE, and protein concentration was checked using Bradford’s method.

### SDS-PAGE and western blot analysis

The expression of the recombinant VEGF_165_ protein in *E. coli* BL21 cells was evaluated by SDS-PAGE and Western Blot. The total protein extract (30 µg) and the purified protein were mixed to loading buffer, heated for 5 min and applied on the gel. 15% SDS-PAGE electrophoresis was conducted in buffer (25 mM Tris; 250 mM Glycine; 1‰ SDS) for 2 h. Separated proteins were directly electro-blotted onto a nitrocellulose membrane in buffer (39 mM Glycine; 48 mM Tris; 0.037% SDS; Methanol 20%) for 1 h at constant voltage (15 V). The membrane was stained with Ponceau S then distained using bi-distilled water, to verify protein-transfer efficiency. The membrane was blocked for 1 h at room temperature with 5% skim milk in phosphate-buffered saline (0.9% NaCl in 10 mM phosphate buffer, pH 7.4) with Tween-20. Immuno-blotting was carried out by incubating the membrane with primary antibody Anti-His Sigma-Aldrich diluted to 1:5000; then with the appropriate Horseradish Peroxidase conjugated secondary antibody diluted to 1:5000. Peroxidase activity was detected using the Amersham enhanced chemo-luminescence system and autoradiography or densitometric analysis performed by the Versadoc MP4000 imaging system (Bio-Rad).

### In vitro endothelial cell proliferation assay

Human umbilical vein endothelial cells (HUVEC) were maintained in RPMI 1640 medium supplemented with 10% fetal calf serum (FCS) in a humidified incubator with 5% CO_2_. Cells were seeded at a density of 5000 cells per well and allowed to grown over-night at 37 °C in a 96-well tissue culture plates until reaching a pre-established confluence.

Various concentrations of recombinant human VEGF_165_ (200 and 500 ng) were added and incubated with HUVEC cells for 72 h. Four duplicate wells were set up for each condition and three independent assays were performed. The proliferation of endothelial cells was evaluated with the MTT test; the treated cells were incubated with 0.5 mg/ml MTT for 2 h at 37 °C. Culture medium was removed carefully from each well and 100 µl of DMSO was added. The plate was then gently agitated until the color reaction was uniform and OD_560nm_ was measured using a microplate reader.

### Chicken chorioallantoic membrane assay

Chick embryos from 3-day-old eggs were opened and placed in double Petri dishes with added water to maintain eggs humidified. After 5 days at 37 °C, filter paper disks (diameter 6 mm) soaked in buffer (0.9% NaCl), 200 ng and 500 ng of recombinant human VEGF_**165**_ were applied on the chicken chorioallantoic membrane (CAM). After 48 h, spontaneous and induced angiogenesis were observed and photographed with a digital camera at 10× magnification. The response was quantified by scoring the extent of vascularization using the software program ImageJ.

### Wound-healing migration assay

Human umbilical vein endothelial cells were cultivated at 37 °C in 48-well plates in RPMI 1640 medium supplemented with 10% fetal calf serum (FCS) and maintained overnight in a humidified incubator (5% CO_2_). Cells were seeded at a density of 5000 cells per well. The next day, monolayers created were carefully scratched using a 20-μl microtip. The cellular debris was subsequently removed by washing with PBS. The cells were thereafter treated with or without recombinant VEGF_165_ (200 ng) in serum free RPMI medium for an additional 12 h. Cell images for each condition were taken with a digital camera connected to an inverted microscope LEICA (×10 objective). The software program ImageJ was used to determine the percentage of wound healing for each condition.

### Statistical analysis

Data is presented as the mean ± SEM of five independent experiments. Statistical significance was analyzed using unpaired Student’s *t* test using STATISTICA 6. p < 0.05 was considered statistically significant and is indicated with asterisks over the value (**p < 0.05 and ***p < 0.001).

## Results

### Amplification of the VEGF splices variants

**Fig. 1:**
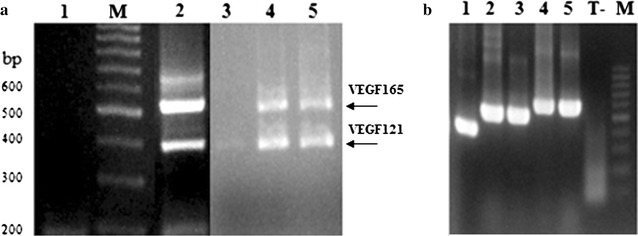
Analysis of electrophoretic profiles. **a** Agarose gel profile of products resulting from PCR amplification of the VEGF transcripts present in tumor tissue: human breast cancer cell line MCF7 (*lane 2*), human colorectal cancer cells (*lane 4*) and human breast cancer cells (*lane 5*) and in distant-tumor tissue (*lane 3*) *M* molecular-mass marker (100 pb DNA ladder; Fermentas); *lane 1* PCR negative control (sample without DNA). **b** Agarose gel showing PCR products from amplification of the positive clones obtained after ligation to pGEMT easy vector. *Lane 1* VEGF_121_, *lane 2/3* VEGF_165_, *lane 4/5* VEGF_189_, T-: PCR negative control (sample without DNA)

Total RNA was extracted from four cell types: the MCF7 cell line, two tumoral biopsies (breast and colorectal cancer) and one adjacent normal tissue from a colorectal cancer patient. The relative abundance of the various VEGF splice variants was determined by RT-PCR using cDNA available in our laboratory. VEGF_165_ and VEGF_121_ were the major variants expressed followed by VEGF_189_ (Fig. 1a). The obtained result is in keeping with findings of various studies which showed that VEGF_165_ VEGF_121_ and VEGF_189_ were routinely the most expressed. Our results showed that only VEGF_121_ was weakly expressed in distant normal tissue whereas a high level expression of VEGF_121_ and VEGF_165_ in tumour tissues was observed (Fig. 1a). In this context, it was reported that VEGF_121_ appeared to be mostly expressed in normal tissue. It was also found that in colorectal tumours, VEGF_121_ expression was similar in both normal and tumour tissue, whereas VEGF_165_ was detected at higher level in tumour tissue (Cressey et al. 2005).

The PCR product mixture obtained after amplification of the MCF7 cDNA was ligated into pGEMT easy vector and cloned in *E. coli* Top10. Several clones were analysed by PCR using the same primers (Fig. 1b) and subsequently sequenced. As expected, the three types of VEGF are represented in the different clones and their sequences were completely identical to the published ones, i.e. the VEGF_165_ with accession number NM_001287044.1 (Piotrowski et al. 2015).

### Heterologous expression VEGF_165_using the pET-21a(+) vector

It should be recalled that the recombinant VEGF_165_ is produced here as a His-Tagged fusion protein. The conditions of culture and induction were optimized at different levels: the culture medium (LB or 2YT), the inducer concentration (0.4 and 1 mM IPTG), the post-induction temperature (25, 30 or 37 °C) and the duration of the culture post-induction.

**Fig. 2:**
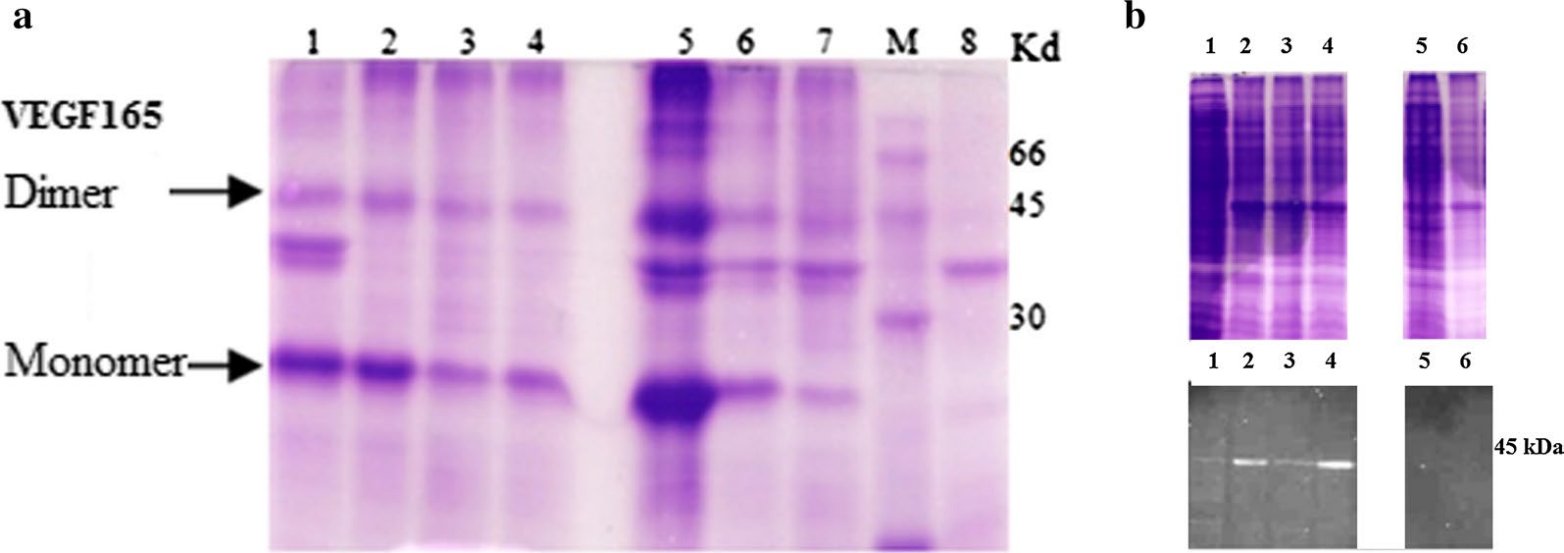
Effect of culture conditions on the expression of VEGF_165_ in the pellet (insoluble) and the supernatant (soluble) of centrifuged crude lysates. **a** Insoluble VEGF_165_ expression at different induction temperature, different IPTG concentration and different medium. *Lanes 1–3*: insoluble VEGF_165_ expression induced with 0.4 mM IPTG in 2YTA medium at the temperature 25, 30 and 37 °C respectively. *Lane 4* insoluble VEGF_165_ expression induced with 1 mM IPTG at 37 °C in 2YTA medium. *Lanes 5–7* insoluble VEGF_165_ expressed in LBA medium and induced with 0.4 mM IPTG at 25, 30 and 37 °C respectively. *Lane M* protein marker (GE Healthcare UK limited). *Lane 8* empty pET 21a vector lysate (control). **b** SDS-PAGE (*top*) and Western blot (*bottom*) analysis of the soluble VEGF165 expression at different induction temperature, different IPTG concentration and different medium. *Lane 1–3* soluble VEGF_165_ expression induced with 0.4 mM IPTG in 2YTA medium at the temperature 25, 30 and 37 °C respectively. *Lane 4* soluble VEGF_165_ expression induced with 1 mM IPTG at 37 °C in 2YTA medium. *Lanes 5–6* soluble VEGF_165_ expressed in LBA medium and induced with 0.4 mM IPTG at 25 and 37 °C respectively

The optimal culture conditions during the induction phase of the recombinant VEGF_165_ were the following: 1 mM of IPTG as inducer in 2YT medium, at 37 °C for 20 h induction time (Fig. 2a). Expression was verified by SDS-PAGE and western blot (Fig. 2b), showing that after IPTG induction for 20 h and only in the 2YT medium, a protein was over-expressed. It migrated, under reducing condition, with an apparent molecular weight of 23 kDa. This band was immuno-recognized by the anti-His-tag antibody. This antibody was also able to detect the homodimeric form of the VEGF_165_.

### Optimization of the VEGF_165_ protein extraction

**Fig. 3:**
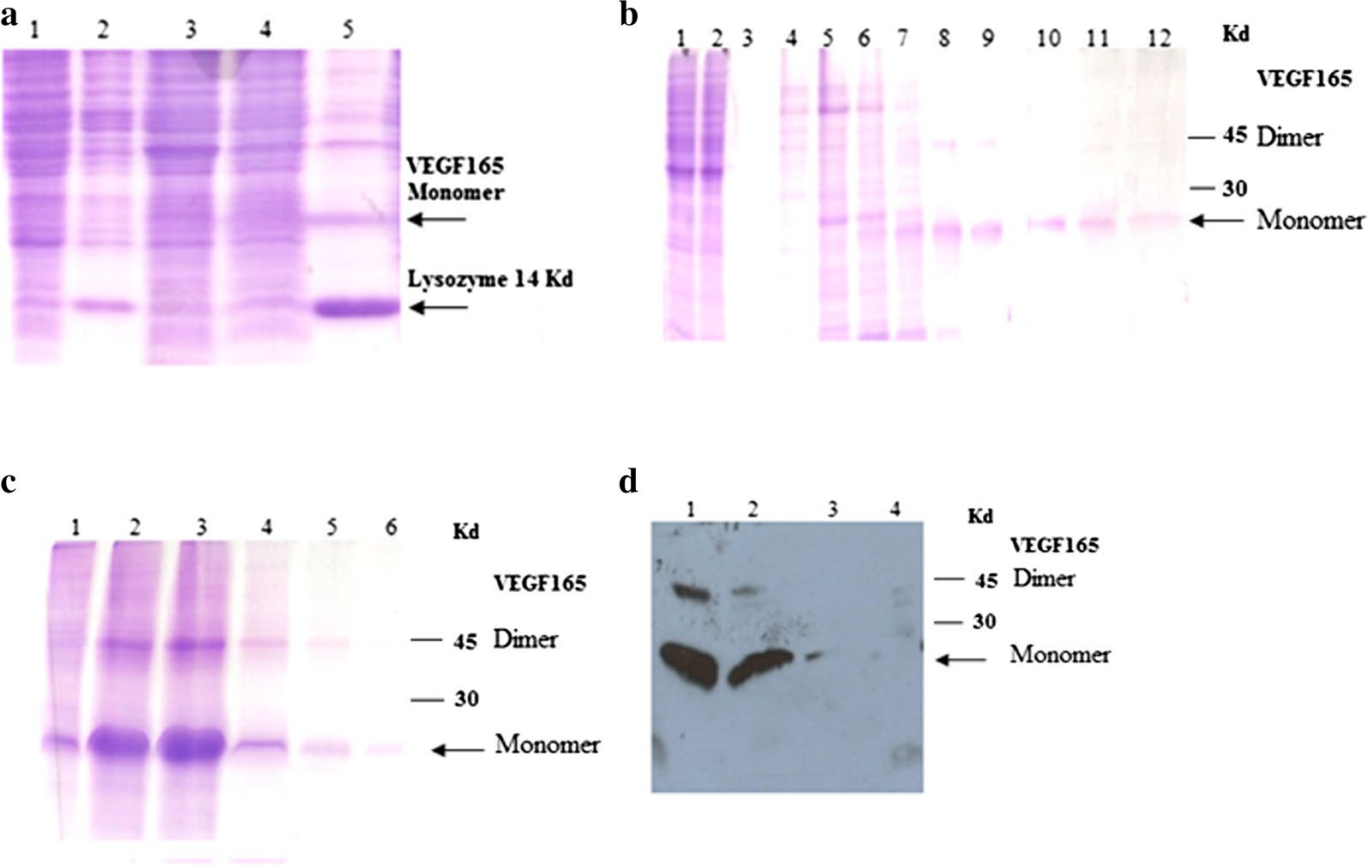
VEGF_165_ expression and purification by SDS-PAGE analysis under optimal condition. **a** VEGF_165_ protein extraction. *Lane 1–5* corresponds respectively to total protein extracted from conditions *M1* Alumina treatment, *M2* lysozyme treatment, *M3* sonication in PBS, *M4* sonication in lysis buffer, *M5* lysozyme treatment followed by sonication in lysis Buffer treatment. Lysozyme and VEGF_165_ are indicated. **b** Soluble VEGF_165_ Purification. *Lane 1* soluble cell lysate before purification; *lane 2* flow-through; *lanes 3–12*: eluted fractions from His-trap column under reducing conditions. **c** SDS-PAGE analysis of the eluate from the refolded IB. *Lane 1* refolded VEGF_165_ before purification; *lane 2–6* eluted fractions with 250 mM Imidazole. **d** Western blot analysis of the VEGF_165_ purification. *Lane 1* eluate from the refolded VEGF_165_; *lane 2* eluate from the soluble VEGF_165_ at 250 mM Imidazole; *lane 3* eluate from the soluble VEGF_165_ at 350 mM; *lane 4* empty pET21a vector lysate

We aimed to compare different extraction protocols of recombinant VEGF. Figure 3a shows that when only sonication was applied, the recombinant protein is faintly seen while it was almost absent in M1 and M2 (using only alumina or lysozyme). The yield of the recombinant VEGF_165_ protein, estimated by SDS-PAGE and Western Blot, was higher when using subsequently lysozyme and sonication treatments, compared to all other methods. The most interesting result in this condition concerned the “clarification” of the majority of the bacterial background, leaving almost only the lysozyme and the recombinant VEGF_165_ and other faint contaminant proteins (Fig. 3a, lane 5).

### VEGF_165_ purification using Nickel-affinity chromatography

Being His-Tagged, the VEGF_165_ was purified on a His Trap column. The total protein extract, from the best method M5, was applied to the column and the fractions were eluted using Imidazole gradient (10–500 mM) and analyzed on 15% SDS-PAGE (Fig. 3b). The VEGF_165_ protein was eluted at 250 mM Imidazole. Western Blot analysis confirmed that this purified protein corresponded to VEGF_165_ (Fig. 3d, lane 2).

### VEGF_165_ recovery from inclusion bodies

Solubilisation and refolding operations are the most important steps that could efficiently convert aggregated protein to bioactive form. In our study, a simple solubilisation method was performed using a high concentration of urea (8 M) and 1% of Triton X-100 to solubilize the pellet. To obtain reduced state of the cysteine residues, β-mercaptoethanol was used as reducing agent. 1 mM EDTA was also added to the solubilisation buffer to prevent metal-catalysed air oxidation of cysteine residues. Thereafter, an elaborate method of proteins aggregate refolding is needed to ensure a good amount of the bioactive VEGF_165_. Therefore, step-wise dialysis was used for the renaturation of the recombinant protein. The gradual removal of the denatured reagent is the most important step as to increase the refolding efficiency of the denatured VEGF_165_. Figure 3c shows that after renaturation, VEGF_165_ was successfully refolded, facilitating thereby its purification. Under reducing conditions, the molecular weight of the refolded VEGF_165_ was 23 kDa. Similarly to the soluble VEGF_165_, we found that elution with 250 mM Imidazole resulted in an increased quantity and purity of the recombinant protein. Both the monomer and the dimer were efficiently eluted.

Finally, we compared the eluted fraction at 250 mM Imidazole from the soluble VEGF_165_ to the refolded VEGF_165_; Fig. 3d shows that they behave similarly by the western blot analysis. The batch fermentation process yields approximately 1.5 mg/l of purified VEGF_165_ from both supernatant and inclusion bodies.

### In vitro HUVEC cells proliferation assay

**Fig. 4:**
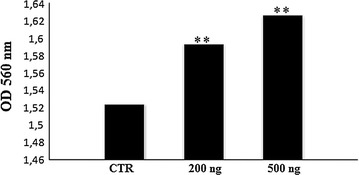
Effect of VEGF_165_ on HUVEC cells proliferation. HUVEC cells were grown to confluence and then incubated with serum free RPMI medium (used as control), 200 and 500 ng of recombinant VEGF_165_. Significant differences: ** means p < 0.05

To examine whether the recombinant human VEGF_165_ was able to induce proliferation of HUVEC cells, we performed MTT experiment with 200 ng and 500 ng of VEGF_165_. Figure 4 showed that pretreated cells resulted in a dose dependent activation of HUVEC cells proliferation. As expected, treatment with 500 ng of VEGF_165_ showed significant cell growth activation when compared to the untreated cells. VEGF stimulation enhanced significantly (p ˂ 0.05) HUVEC cells proliferation.

### Chicken chorioallantoic membrane assay

**Fig. 5:**
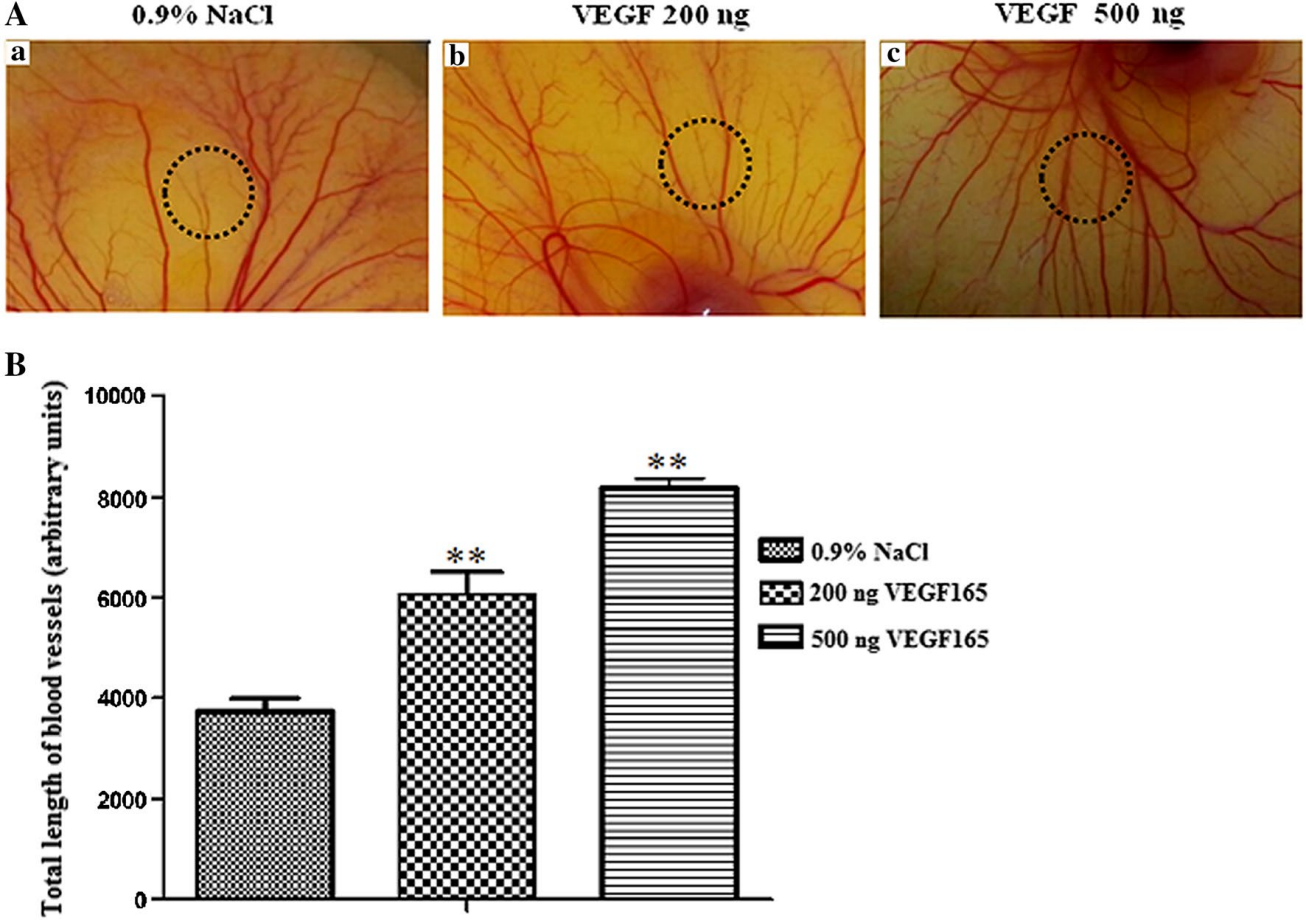
Recombinant VEGF_165_ induces ex vivo angiogenesis. **A** The CAM models were prepared using 8-day-old chick embryos treated as described in method section. *Dark circles* represent location of applied disks. Filter disks were soaked in (*a*) 0.9% NaCl; (*b*) 200 ng of recombinant VEGF; (*c*) 500 ng of recombinant VEGF; after incubation for 48 h, CAMs were photographed with a digital camera. Each group contained four CAMs and the experiment was repeated three times. **B** The quantitative measurement of total vessel length was performed on 50% of the total CAM surface treated in the absence or in the presence of recombinant VEGF. Significant differences: ** means p < 0.05

To further characterize the pro-angiogenic properties of recombinant VEGF_165_, we performed ex vivo angiogenesis using chick chorioallantoic membrane (CAM) assays. Upon dissection of the CAM of 8-day-old chick embryos, filter paper disks soaked in buffer (0.9% NaCl) used as control, 200 and 500 ng of recombinant VEGF were applied on the CAM. The spontaneous angiogenesis in CAM was observed after 48 h. As illustrated in Fig. 5A, recombinant VEGF induced remarkably the number of new capillaries and branching vessels in the CAM. Furthermore, an increase in the vascular density could be observed. Quantification shows that the total vessel length was induced by 50 and 100% by 200 and 500 ng doses, respectively (Fig. 5A, b and c), compared with the untreated conditions (Fig. 5A, a).

### In vitro scratch wound assay

**Fig. 6:**
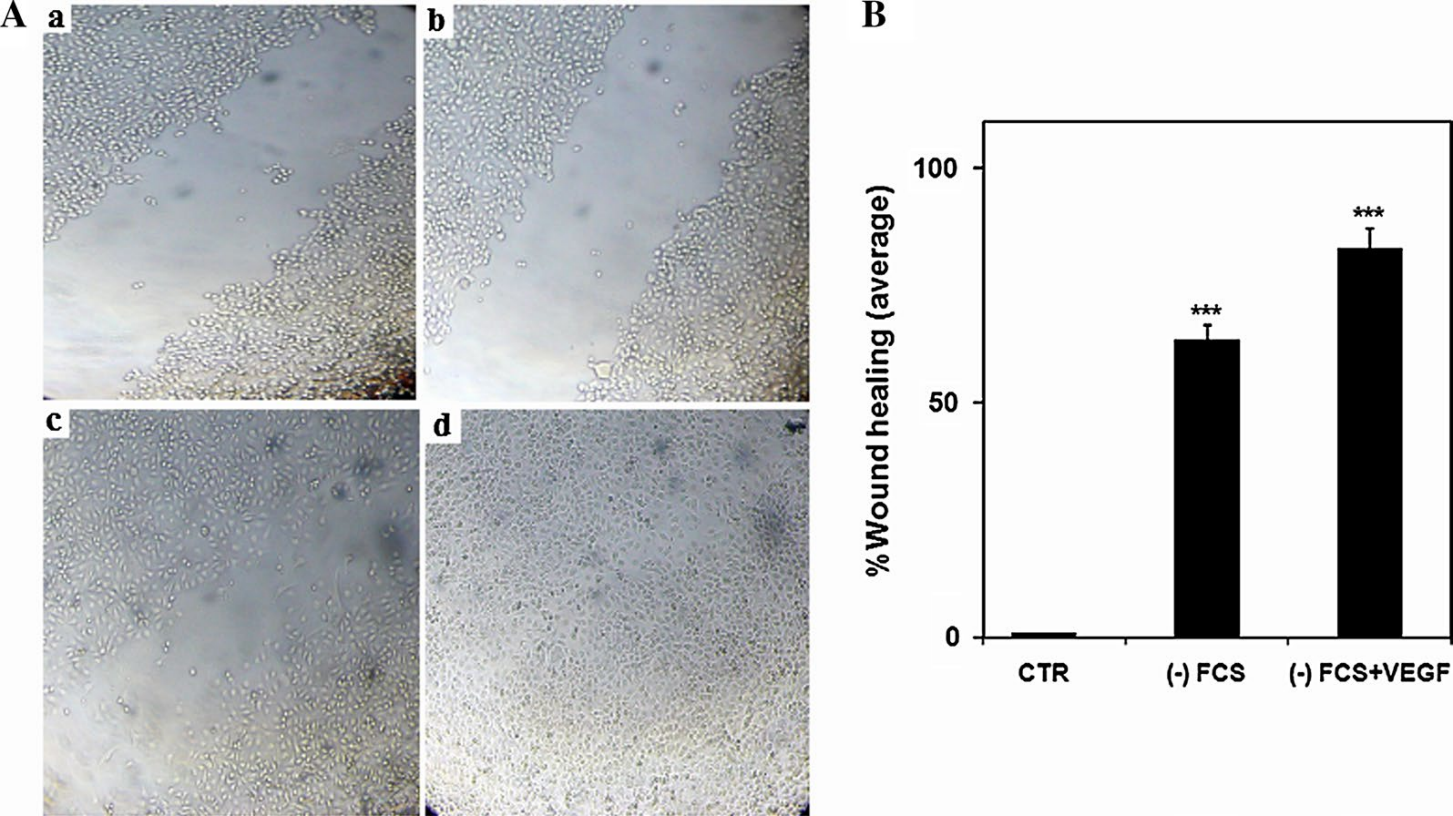
Cell migration in response to VEGF_165_. **A** *a* The free untreated cell area at T0 h. *b* The gap area of induced cells at T0 h. *c* untreated cells at T12 h. *d* The cell migration in response to treatment with 200 ng VEGF_165_ at T12 h. **B** Histogram showing the percentage of wound healing for each condition. Significant differences: *** means p < 0.001

Because endothelial cell migration is very important in VEGF-associated wound healing, we performed in vitro scratch assay employing HUVEC cells. In order to evaluate the functionality of the recombinant VEGF_165_, cells were cultured for 12 h in serum free RPMI medium containing or not 200 ng of VEGF_165_. Compared with T12h treated cells with 200 ng VEGF_165_, the non-treated HUVEC cells did not significantly migrate into the scratched site under any growth factor stimulation. Images were analysed for the gap area over time (T0 h: Fig. 6Aa, Ab and T12h: Fig. 6Ac, Ad). We showed a high statistically significant difference between untreated HUVEC cells and those treated with 200 ng VEGF_165_. Our finding shows that HUVEC cells migrate into the scratched site under any growth factor stimulation and the wound closure extent to 65% (Fig. 6Ac), while cell migration into the free area induced by 200 ng VEGF_165_ was significantly increased by 15% and the wound closure extent to 80% (Fig. 6Ad, Fig. 6B). The obtained result is in keeping with findings of various studies which showed that VEGF_165_ stimulates endothelial cell migration (Pan et al. 2014; Van der Meer et al. 2010). This confirms that the VEGF produced here is biological active.

## Discussion

The production of VEGF_165_ in significant amounts is an important prerequisite for the search, expansion of promising and effective anti-angiogenic drugs. Many attempts for producing VEGF_165_ in bacterial system have been made. They have mainly focused on the optimization of production conditions such as induction temperature, IPTG concentration and time incubation after induction (Kang et al. 2013). Generally, low temperature ensured the expression of less inclusion bodies and more soluble form of recombinant protein but some reports found that the soluble recombinant VEGF_165_ expression was increased when the cells were incubated at 37 °C (Lee et al. 2011). Interestingly, we showed that the expression of the VEGF_165_ induced with 1 mM IPTG in 2YT medium at 37 °C for 20 h increased the percentage of the soluble protein. In this expression assay, inclusion bodies occurred heavily when the inducing temperature was set at 37 °C. We also showed that VEGF protein was much more expressed in 2YTA than in LBA medium, probably because it was exclusively produced as inclusion bodies in LBA condition.

The common objective of a successful heterologous expression is generally to balance success rates with speed, ease, cost and breadth of use. For this reason, different parameters for VEGF_165_ expression are needed to ensure a high level and a good yield of the recombinant protein. The central interest of many works is to optimize production conditions such as shaking speed, medium, induction temperature, IPTG concentration, but the optimization of the protein extraction method remains a major challenge for a high percentage of the produced protein. It is apparent that the methods described here have, in many instances, to be quite similar especially with use of sonication in lysis buffer to extract target proteins. But combining different protein extraction method is not frequently used. In this study, we found that combining lysozyme treatment and sonication in lysis buffer (M5) could increase the level of the soluble VEGF_165_. More interestingly, the consequence of this method is the important clarification of the protein lysate, thereby facilitating the purification of the VEGF_165_. A single step of purification using affinity chromatography that does not need any organic solvent like acetonitrile was carried out.

Many procedures for producing recombinant human VEGF_165_ in bacterial system were described but resulted in most of cases in the production of insoluble inclusion bodies which represented the primary source of the target protein (Gast et al. 2011). With the fact that 30% of proteins from *E. coli* itself cannot be expressed in soluble form (Gräslund et al. 2008), it is meant that the main limitations of the recombinant protein expression from bacterial cells are the low production levels and low refolding yield of the inclusion bodies, leading to biologically inactive recombinant proteins (Bang et al. 2013).

It was reported that aggregation reactions of different proteins displayed certain common properties. A strong temperature dependence of unfolding enthalpy which increases rapidly with temperature was shown. The use of low temperature during induction phase could increase peptide stability and reduce inclusion bodies formation. In contrast to previous studies (Kim et al. 2007; Zhang et al. 2014) we found that maintaining induction temperature at 37 °C for 20 h led to a low aggregation level of the VEGF_165_ improving its solubility. Nevertheless, our protein was expressed largely in the form of inclusion bodies. The protein aggregates was solubilized using high concentration of Urea while β-Mercaptoethanol was added to split the disulfide bond (that are responsible of the inter-subunit aggregation) and to maintain cysteine residues in a reduced state. The step-wise dialyses used allow protein folding into their native form and decrease sufficiently the denaturant concentration.

This insoluble fraction was therefore subjected to a simple procedure that overcomes the need of multi-days refolding experiments (Lee et al. 2011). Indeed, these authors spent about 7 days of serial dialysis in order to recover a refolded protein while our procedure takes only 48 h.

A single step purification of the refolded VEGF_165_ was thereafter carried out using a Nickel affinity column. Similarly to the soluble VEGF_165_, the refolded protein was eluted at 250 mM Imidazole and detected by anti His-tag antibodies. On the other hand, the function of VEGF in wound repair has been extensively studied. Thereby, VEGF stimulates angiogenesis and also influences wound closure and epidermal repair. Here, the biological activity of the recombinant VEGF_165_ was verified by chicken chrioallantoic membrane assay, scratch wound healing and proliferation assay using HUVEC cells. Thus, VEGF_165_ potency and bioactivity were confirmed by its ability to promote endothelial cell migration and proliferation and to induce new capillaries and branching vessels in the CAM model.

In summary, this study described a simple approach for producing recombinant VEGF_165_ for therapeutic applications. The overall probability of expressing human VEGF successfully depends on the variation of the large-scale production parameters and also protein extraction methods that could increase the yield and the purity of the soluble protein. Protein extraction was a major challenge that could assess the purity of the VEGF_165_ and lead to a simplified purification procedure. Overall, a successfully VEGF_165_ expression in bacterial system within soluble and insoluble fraction with fast and low cost procedure was presented to produce efficiently a functional VEGF protein for therapeutic applications.
